# Supplementary material for: A Critical View of the Application of the APEX Software (Aqueous Photochemistry of Environmentally-Occurring Xenobiotics) to Predict Photoreaction Kinetics in Surface Freshwaters
Source: Molecules. 2019 Dec 18;25(1):9. doi: 10.3390/molecules25010009 (PMC7017383; doi:10.3390/molecules25010009)
Supplement: Supplementary file 1 [file molecules-25-00009-s001.zip › 3.2.4_gcc-4.4.0.zip!/3.2.4_gcc-4.4.0/3.2.4_gcc-4.4.0/doc/octave/html/interpreter/Basic-Matrix-Functions.html]

Basic Matrix Functions - Untitled


Next: Matrix Factorizations,
Previous: Techniques used for Linear Algebra,
Up: Linear Algebra


---

### 18.2 Basic Matrix Functions

— Loadable Function: aa = **balance** (a, opt)  
— Loadable Function: [dd, aa] = **balance** (a, opt)  
— Loadable Function: [d, p, aa] = **balance** (a, opt)  
— Loadable Function: [cc, dd, aa, bb] = **balance** (a, b, opt)  
> Compute `aa = dd \ a * dd` in which `aa` is a matrix whose
> row and column norms are roughly equal in magnitude, and
> `dd` = `p * d`, in which `p` is a permutation
> matrix and `d` is a diagonal matrix of powers of two. This allows
> the equilibration to be computed without roundoff. Results of
> eigenvalue calculation are typically improved by balancing first.
>
> If two output values are requested, `balance` returns
> the diagonal `d` and the permutation `p` separately as vectors.
> In this case, `dd = eye(n)(:,p) * diag (d)`, where `n` is the matrix
> size.
>
> If four output values are requested, compute `aa = cc*a*dd` and
> `bb = cc*b*dd)`, in which `aa` and `bb` have non-zero
> elements of approximately the same magnitude and `cc` and `dd`
> are permuted diagonal matrices as in `dd` for the algebraic
> eigenvalue problem.
>
> The eigenvalue balancing option `opt` may be one of:
>
> `"noperm"`, `"S"`: Scale only; do not permute. `"noscal"`, `"P"`: Permute only; do not scale.
>
> Algebraic eigenvalue balancing uses standard lapack routines.
>
> Generalized eigenvalue problem balancing uses Ward's algorithm
> (SIAM Journal on Scientific and Statistical Computing, 1981).

— Function File: **cond** (a,p)  
> Compute the p-norm condition number of a matrix. `cond (`a`)` is
> defined as `norm (`a`,` p`) * norm (inv (`a`),` p`)`.
> By default p`=2` is used which implies a (relatively slow)
> singular value decomposition. Other possible selections are
> p`= 1, Inf, inf, 'Inf', 'fro'` which are generally faster.
>
>
>
> **See also:** condest, rcond, norm, svd.

— Loadable Function: [d, rcond] = **det** (a)  
> Compute the determinant of a using lapack for full and UMFPACK
> for sparse matrices. Return an estimate of the reciprocal condition number
> if requested.

— Function File: **dmult** (a, b)  
> This function has been deprecated. Use the direct syntax `diag(A)*B`
> which is more readable and now also more efficient.

— Function File: **dot** (x, y, dim)  
> Computes the dot product of two vectors. If x and y
> are matrices, calculate the dot-product along the first
> non-singleton dimension. If the optional argument dim is
> given, calculate the dot-product along this dimension.

— Loadable Function: lambda = **eig** (a)  
— Loadable Function: lambda = **eig** (a, b)  
— Loadable Function: [v, lambda] = **eig** (a)  
— Loadable Function: [v, lambda] = **eig** (a, b)  
> The eigenvalues (and eigenvectors) of a matrix are computed in a several
> step process which begins with a Hessenberg decomposition, followed by a
> Schur decomposition, from which the eigenvalues are apparent. The
> eigenvectors, when desired, are computed by further manipulations of the
> Schur decomposition.
>
> The eigenvalues returned by `eig` are not ordered.
>
>
>
> **See also:** eigs.

— Loadable Function: g = **givens** (x, y)  
— Loadable Function: [c, s] = **givens** (x, y)  
> Return a 2 by 2 orthogonal matrix
> g `= [`c s`; -`s`'` c`]` such that
> g `[`x`;` y`] = [*; 0]` with x and y scalars.
>
> For example,
>
> ```
>           givens (1, 1)
>                =>   0.70711   0.70711
>                    -0.70711   0.70711
> ```

— Function File: [g, y] = **planerot** (x)  
> Given a two-element column vector, returns the
> 2 by 2 orthogonal matrix
> G such that
> y `=` g `*` x and y`(2) = 0`.
>
>
>
> **See also:** givens.

— Loadable Function: [x, rcond] = **inv** (a)  
— Loadable Function: [x, rcond] = **inverse** (a)  
> Compute the inverse of the square matrix a. Return an estimate
> of the reciprocal condition number if requested, otherwise warn of an
> ill-conditioned matrix if the reciprocal condition number is small.
>
> If called with a sparse matrix, then in general x will be a full
> matrix, and so if possible forming the inverse of a sparse matrix should
> be avoided. It is significantly more accurate and faster to do
> y `=` a `\` b, rather than
> y `= inv (`a`) *` b.

— Loadable Function: type = **matrix\_type** (a)  
— Loadable Function: a = **matrix\_type** (a, type)  
— Loadable Function: a = **matrix\_type** (a, 'upper', perm)  
— Loadable Function: a = **matrix\_type** (a, 'lower', perm)  
— Loadable Function: a = **matrix\_type** (a, 'banded', nl, nu)  
> Identify the matrix type or mark a matrix as a particular type. This allows rapid
> for solutions of linear equations involving a to be performed. Called with a
> single argument, `matrix_type` returns the type of the matrix and caches it for
> future use. Called with more than one argument, `matrix_type` allows the type
> of the matrix to be defined.
>
> The possible matrix types depend on whether the matrix is full or sparse, and can be
> one of the following
>
> 'unknown': Remove any previously cached matrix type, and mark type as unknown 'full': Mark the matrix as full. 'positive definite': Probable full positive definite matrix. 'diagonal': Diagonal Matrix. (Sparse matrices only) 'permuted diagonal': Permuted Diagonal matrix. The permutation does not need to be specifically indicated, as the structure of the matrix explicitly gives this. (Sparse matrices only) 'upper': Upper triangular. If the optional third argument perm is given, the matrix is assumed to be a permuted upper triangular with the permutations defined by the vector perm. 'lower': Lower triangular. If the optional third argument perm is given, the matrix is assumed to be a permuted lower triangular with the permutations defined by the vector perm. 'banded' 'banded positive definite': Banded matrix with the band size of nl below the diagonal and nu above it. If nl and nu are 1, then the matrix is tridiagonal and treated with specialized code. In addition the matrix can be marked as probably a positive definite (Sparse matrices only) 'singular': The matrix is assumed to be singular and will be treated with a minimum norm solution
>
> Note that the matrix type will be discovered automatically on the first attempt to
> solve a linear equation involving a. Therefore `matrix_type` is only
> useful to give Octave hints of the matrix type. Incorrectly defining the
> matrix type will result in incorrect results from solutions of linear equations,
> and so it is entirely the responsibility of the user to correctly identify the
> matrix type.
>
> Also the test for positive definiteness is a low-cost test for a hermitian
> matrix with a real positive diagonal. This does not guarantee that the matrix
> is positive definite, but only that it is a probable candidate. When such a
> matrix is factorized, a Cholesky factorization is first attempted, and if
> that fails the matrix is then treated with an LU factorization. Once the
> matrix has been factorized, `matrix_type` will return the correct
> classification of the matrix.

— Built-in Function: **norm** (a, p, opt)  
> Compute the p-norm of the matrix a. If the second argument is
> missing, `p = 2` is assumed.
>
> If a is a matrix (or sparse matrix):
>
> p = `1`: 1-norm, the largest column sum of the absolute values of a. p = `2`: Largest singular value of a. p = `Inf` or `"inf"`: Infinity norm, the largest row sum of the absolute values of a. p = `"fro"`: Frobenius norm of a, `sqrt (sum (diag (`a`' *` a`)))`. other p, p `> 1`: maximum `norm (A*x, p)` such that `norm (x, p) == 1`
>
> If a is a vector or a scalar:
>
> p = `Inf` or `"inf"`: `max (abs (`a`))`. p = `-Inf`: `min (abs (`a`))`. p = `"fro"`: Frobenius norm of a, `sqrt (sumsq (abs (a)))`. p = 0: Hamming norm - the number of nonzero elements. other p, p `> 1`: p-norm of a, `(sum (abs (`a`) .^` p`)) ^ (1/`p`)`. other p p `< 1`: the p-pseudonorm defined as above.
>
> If `"rows"` is given as opt, the norms of all rows of the matrix a are
> returned as a column vector. Similarly, if `"columns"` or `"cols"` is passed
> column norms are computed.
>
>
>
> **See also:** cond, svd.

— Function File: **null** (a, tol)  
> Return an orthonormal basis of the null space of a.
>
> The dimension of the null space is taken as the number of singular
> values of a not greater than tol. If the argument tol
> is missing, it is computed as
>
> ```
>           max (size (a)) * max (svd (a)) * eps
> ```

— Function File: **orth** (a, tol)  
> Return an orthonormal basis of the range space of a.
>
> The dimension of the range space is taken as the number of singular
> values of a greater than tol. If the argument tol is
> missing, it is computed as
>
> ```
>           max (size (a)) * max (svd (a)) * eps
> ```

— Loadable Function: **pinv** (x, tol)  
> Return the pseudoinverse of x. Singular values less than
> tol are ignored.
>
> If the second argument is omitted, it is assumed that
>
> ```
>           tol = max (size (x)) * sigma_max (x) * eps,
> ```
>
> where `sigma_max (`x`)` is the maximal singular value of x.

— Function File: **rank** (a, tol)  
> Compute the rank of a, using the singular value decomposition.
> The rank is taken to be the number of singular values of a that
> are greater than the specified tolerance tol. If the second
> argument is omitted, it is taken to be
>
> ```
>           tol = max (size (a)) * sigma(1) * eps;
> ```
>
> where `eps` is machine precision and `sigma(1)` is the largest
> singular value of a.

— Loadable Function: c = **rcond** (a)  
> Compute the 1-norm estimate of the reciprocal condition as returned
> by lapack. If the matrix is well-conditioned then c will be near
> 1 and if the matrix is poorly conditioned it will be close to zero.
>
> The matrix a must not be sparse. If the matrix is sparse then
> `condest (`a`)` or `rcond (full (`a`))` should be used
> instead.
>
>
>
> **See also:** inv.

— Function File: **trace** (a)  
> Compute the trace of a, `sum (diag (`a`))`.

— Function File: [r, k] = **rref** (a, tol)  
> Returns the reduced row echelon form of a. tol defaults
> to `eps * max (size (`a`)) * norm (`a`, inf)`.
>
> Called with two return arguments, k returns the vector of
> "bound variables", which are those columns on which elimination
> has been performed.
